# A Case of Femoral Arteriovenous Fistula Causing High-Output Cardiac Failure, Originally Misdiagnosed as Chronic Fatigue Syndrome

**DOI:** 10.1155/2014/510429

**Published:** 2014-05-20

**Authors:** J. Porter, Q. Al-Jarrah, S. Richardson

**Affiliations:** Department of Vascular Surgery, University Hospital of South, Manchester, Southmoor Road, Manchester M23 9LT, UK

## Abstract

Percutaneous arterial catheterisation is commonly undertaken for a range of diagnostic and interventional procedures. Iatrogenic femoral arteriovenous fistulas are an uncommon complication of these procedures. Most are asymptomatic and close spontaneously, but can rarely increase in size leading to the development of symptoms. We report a case of an iatrogenic femoral arteriovenous fistula, causing worsening congestive cardiac failure, in a 34-year-old marathon runner. This was originally diagnosed as chronic fatigue syndrome. Following clinical examination, duplex ultrasound, and CT angiography a significant arteriovenous fistula was confirmed. Elective open surgery was performed, leading to a dramatic and rapid improvement in symptoms. Femoral arteriovenous fistulas have the potential to cause significant haemodynamic effects and can present many years after the initial procedure. Conservative, endovascular, and open surgical management strategies are available.

## 1. Introduction

Percutaneous arterial catheterisation, using the femoral artery, is commonly undertaken for a range of diagnostic and interventional procedures. Potential complications include puncture site haemorrhage, pseudoaneurysm formation, vessel dissection, distal thrombosis, and rarely arteriovenous fistulas (AVFs) formation [1, 2]. Arteriovenous fistulas can cause significant complications including congestive cardiac failure and threatening of lower limb circulation.

The incidence of postcatheterisation AVFs ranges from 0.006 to 0.86% [2, 3]. AVFs in the groin frequently result from low groin punctures. At this level, haemostasis by compression is difficult to achieve and the anatomical positions of the common femoral vein lying almost posterior to the superficial femoral artery increase the likelihood of AVF formation [4].

We present a case of iatrogenic femoral arteriovenous fistula, leading to progressively worsening congestive cardiac failure, originally diagnosed as chronic fatigue syndrome, in a young woman.

## 2. Case Presentation

A 34-year-old woman underwent percutaneous closure of a patent foramen ovale, using the right femoral artery, three years earlier. During her initial procedure, significant bruising around the puncture site and wound infection was noted.

She presented several years later with progressively worsening fatigue, lethargy, originally diagnosed as chronic fatigue syndrome. As her symptoms deteriorated, she developed exertional dyspnoea. This had a significant effect on her quality of life, causing her to cease employment and take long-term sick leave. Aside from her original cardiac surgery, she had no other significant past medical history.

Routine blood tests did not reveal any evidence of renal disease and a transthoracic echocardiogram demonstrated good left ventricular systolic function with no significant valvular disease.

She incidentally noted a “buzzing” sensation in her right groin and further evaluation by her general practitioner revealed a palpable thrill leading to referral to our vascular surgery department.

**Figure 1:**
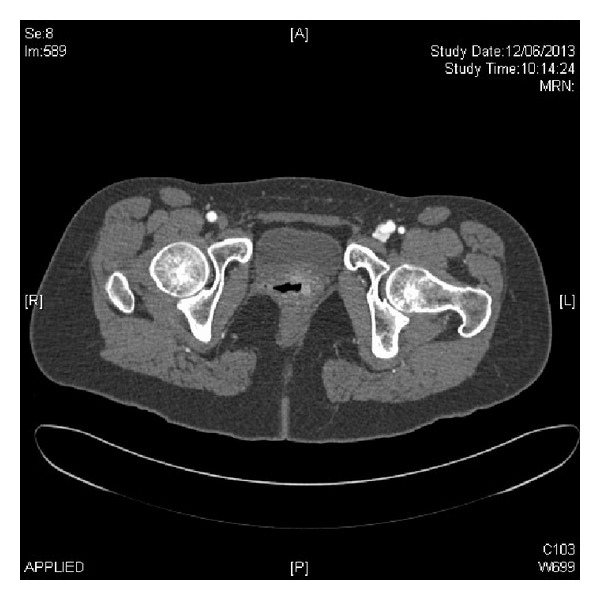
A CT angiogram demonstrating the abnormal contrast flow between the SFA and CFV, diagnosing the arteriovenous fistula.

Clinical assessment revealed a palpable thrill and a continuous audible bruit in the affected groin. Duplex ultrasound demonstrated an AVF between the superficial femoral artery (SFA) and common femoral vein (CFV), 1 cm below the common femoral artery bifurcation. Peak systolic velocity at the fistula site was measured at 1076 cm/s. Subsequent CT imaging confirmed the presence of an AVF, with rapid early venous filling of the CFV with associated dilation of the iliac vein and inferior vena cava (Figure 1).

**Figure 2:**
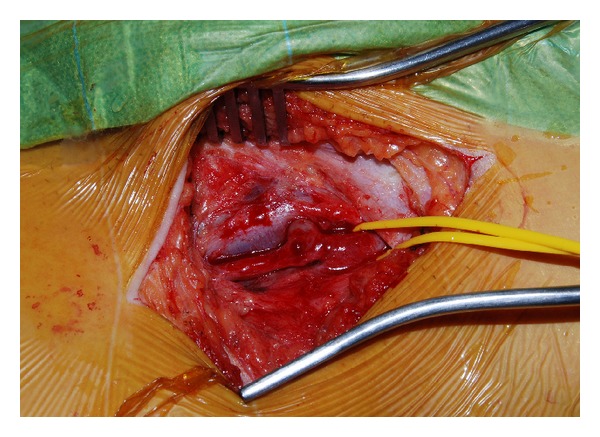
An intraoperative photo demonstrating the AV fistula between SFA and CFV.

Given the symptomatic nature of the lesion, surgery was undertaken. Surgical exploration revealed a well-developed connection between SFA and CFV (Figure 2). The SFA and CFV were separated after arterial and venous control was established. The fistula was excised and the CFV closed with a running nonabsorbable suture. The CFA was closed using a vein patch, to avoid possible future arterial stenosis from primary closure.

Postoperatively, right lower limb pulses are present and the groin thrill had ceased. A prompt recovery was made with immediate improvement in symptoms. At six-week clinic follow-up, her symptoms of exertional dyspnoea had resolved, generalised fatigue improved, and she has begun to exercise once again.

## 3. Discussion

AVFs following percutaneous catheterisation are usually small and asymptomatic and close spontaneously over several months. If they persist, a well-developed fistula tract can increase in size leading to significant shunting of blood from the arterial to venous system. The increased venous return can eventually lead to the development of a high-output cardiac failure state [5].

Diagnosis of AVFs can be made by history and clinical examination and supported by imaging investigations. Possible symptoms include swelling, thrill, bruit, and pulse deficit in the affected limb. Systemic symptoms, from haemodynamically significant lesions, include dyspnoea, especially on exercise, lethargy, and leg swelling [5].

Investigations include the Doppler ultrasound (duplex ultrasonography), CT angiography, and MR imaging as well as conventional catheter angiography.

Watchful waiting for spontaneous closure with serial ultrasound follow-up and a trial of ultrasound-guided compression are appropriate first-line treatment options, although less likely to be successful for patients taking anticoagulants [6]. Surgical repair remains the gold standard, consisting of identification of the fistula tract and resection followed by primary or patch closure repair. Complications include significant haemorrhage from venous hypertension of the arterialised vein and groin infection.

Endovascular modalities include covered vascular stents and coil embolisation. However, closure proximity to the bifurcation of the CFA precluded its use in this instance.

In our presented case, this young lady had no other demonstrable cause for her significant symptoms, and her prompt improvement following surgery confirmed the AVF as the cause. Other clinicians should be mindful of the profound haemodynamic effects of these types of fistula, which can cause symptoms which we described in an otherwise healthy young athlete.

Risk factors for the development of AVF include high heparin dosage, low groin puncture, warfarin therapy, arterial hypertension, and female gender [1]. Other possible risk factors include operator technique, repeated attempts to gain vascular access, excess haemorrhage, and failure to control haemostasis after catheter sheath removal [4].

We would advocate routine use of ultrasound-guided groin arterial punctures to minimise the risk of femoral AVF development.
